# Knowledge, attitudes and prevention practices regarding HIV/AIDS among barbers in Ho municipality, Ghana

**DOI:** 10.1080/17290376.2021.1883101

**Published:** 2021-02-28

**Authors:** Mercy Demaris Quarm, Jacqueline Mthembu, Khangelani Zuma, Elvis Enowbeyang Tarkang

**Affiliations:** a University of Health and Allied Sciences, Ho, Ghana; b Human Sciences Research Council, Pretoria, South Africa

**Keywords:** Knowledge, attitudes and prevention practice, HIV/AIDS, barbers, Ho, Ghana

## Abstract

The barbing industry poses particular public health risks if it is not conducted in a safe and hygienic manner. These risks can lead to the transmission of infectious diseases like the Human Immunodeficiency Virus (HIV) to the barbers or their clients. This study investigated the knowledge, attitudes and prevention practices regarding HIV transmission among barbers in the Ho Municipality. A descriptive cross-sectional design was employed. A pretested structured questionnaire was administered to a consented sample of barbers sampled using a multistage random sample design. Descriptive and inferential statistics were performed using Stata version 14.0 software programme where 0.05 level was used as a measure of significance. The knowledge level of the barbers regarding HIV/AIDS was inadequate (63.6%). Knowledge was significantly associated with work experience [AOR = 13.56 (95% CI: 2.73–67.25); *p* = 0.001], with attitude [AOR = 4.07 (95% CI: 1.27–13.08); *p* = 0.018], with level of education [AOR = 10.22 (95% CI: 2.24–46.64); *p* = 0.003], with marital status [AOR = 0.07 (95% CI: 0.01–0.50); *p* = 0.008] and with number of clients per day [AOR = 0.13 (95% CI: 0.03–0.52); *p* = 0.004]. The attitude of barbers was also inadequate (58.7%). Attitude was significantly associated with the mode of learning the barbing profession [AOR = 0.32 (95% CI: 0.11–0.89); *p* = 0.029], and with level of knowledge [AOR = 5.48 (95% CI: 2.01–14.93); *p* = 0.001]. Majority of the participants exhibited poor prevention practices regarding HIV/AIDS (87.6%). Prevention practice was significantly associated with work experience [AOR = 24.92 (95% CI: 2.08–297.86); *p* = 0.011] and with level of knowledge [AOR = 12.57 (95% CI: 1.35–116.86); *p* = 0.026]. The barbers in Ho exhibited inadequate knowledge and attitude regarding HIV/AIDS, and also manifested poor prevention practices regarding HIV/AIDS. Programmes aimed at improving the knowledge, attitudes and prevention practices should be implemented among barbers, with focus on those without any formal education, those with less than five years work experience and those with more than ten clients a day.

## Background

HIV/AIDS still remains a major public health problem all over the world, particularly in sub-Saharan Africa (SSA) where it has caused incalculable human suffering, social and cultural disruption and huge economic losses, as UNAIDS estimates 36.7 million people living with HIV (PLHIV) globally, and 25.6 million in SSA (UNAIDS, 2016). The number of PLHIV in Ghana as estimated by UNAIDS is 274,600, with deaths due to AIDS being 9,200, and an HIV prevalence of 2.4% (UNAIDS, 2016).

HIV-infected individuals may remain asymptomatic for years; thus, patients with HIV infection may be unaware of the infection and may spread it to others (Ataei, Shirani, Alavian, & Ataie, 2013). HIV can be transmitted through unsafe use of therapeutic injections, blood transfusions, mother-to-child transmission (MTCT), unsafe sexual practices and some beauty treatments like tattooing, piercing, pedicure and barbershop shaving with unsterilised instruments (Patel et al., 2015).

Prevention is the only viable way to control HIV spread, as there is no cure for the infection presently. In Ghana, efforts have focused mainly on prevention of HIV through sexual intercourse, blood transfusion and MTCT. This must have been informed by the fact that more than ninety percent of HIV transmission occurs through the combination of these routes (UNFPA, 2014). However, HIV transmission through sharing of non-sterile sharp instruments such as those used for barbing, circumcision, facial scarification, incision, tattooing, ear perforation, injections and acupuncture have always been vaguely classified as ‘others’ and given less attention in the campaign against the spread of HIV.

Barbers are workers that undertake skin-piercing practices involving re-useable sharp instruments, which present risks of transmission of HIV and other blood-borne pathogens from one client to the other. Though barbers do not carry out procedures that deliberately penetrate the skin, the procedures can inadvertently damage the skin through abrasion or minor accidental cuts (Patel et al., 2015). A simple nick caused by a clipper or razor blade is enough for HIV infection to occur (Humphries, 2000). In most cases, this sharp equipment are not often sterilised, thus exposing clients to blood borne pathogens, including HIV (Biadgelegn et al., 2012).

Due to the lipid envelope that protects HIV from dehydration, the virus can survive on the surfaces of barbing instruments for a period long enough for transmission to occur, particularly in commercial barbing shops (Khandait, Ambadekar, & Vasudeo, 1999). Therefore, barbers can play a role in the spread of this infection. During barbing, there are haircuts and shavings, which may accidentally expose barbers to client’s blood and transmit infection to them or transmit the infection from one client to another. In many parts of Africa, the widespread cultural practice of barbing at a shop or roadside is an underestimated route of blood-borne viral disease transmission.

Proper, effective and consistent decontamination of barbing instruments is important in preventing HIV transmission in barbing shops. Surgically, barbing instruments are semi-critical instruments that come into contact with damaged non-intact skin and require at least intermediate-level disinfection to make them safe (Gardner, 2002). Methods and agents that have been designed to inactivate other viruses such as hepatitis B, are also effective for HIV. These agents include alcohol (ethanol, isopropyl), chlorine (Sodium hypochlorite), phenolic compounds, quaternary ammonium compounds, iodine and iodophore. HIV on barbing instruments can also be inactivated by using sterilising agents such as flame, dry heat, cream and ultraviolet light (CDC, 2008).

Barbers tend to concentrate their attention on providing decoration, air conditioning, sound system and also availability of television in their shops rather than on the risk factors associated with their profession (Shapiro & Margolis, 2008). The concept of universal precaution considers all blood and body fluids to be potentially infectious and all invasive instruments to be potentially contaminated if already used (Dongdem et al., 2012). The responsibility to keep instruments free of infective agents lies on the barbers.

There is little research regarding knowledge, attitudes and prevention practices of barbers in Ghana with respect to the transmission of HIV (Addo, Yawson, Addo, Dornoo, & Seneadza, 2014), but none in the Volta region. Ho, the Capital of the Volta region of Ghana, has an HIV prevalence of 2.6%, which is above the national prevalence (NACP, 2018). This study was therefore conducted to assess the knowledge, attitudes and prevention practices regarding HIV/AIDS among barbers in the Ho Municipality, Ghana.

## Methods

### Study site description

The Ho Municipality located in the Volta region of Ghana is located between latitudes 6° 20°″ N and 6° 55°″ N and longitudes 0° 12°″ E and 0° 53°″ E. The municipality shares boundaries with Adaklu and Agortime-Ziope Districts to the South, Ho West District to the North-West and the Republic of Togo to the East. Its total land area is 2361 square kilometers, thus representing 11.5 percent of the region’s total land area. The population of Ho Municipality according to the 2010 Population and Housing Census is 177,281 representing 8.4 percent of the region’s total population. Females constitute 52.7 percent and males represent 47.3 percent. About 62 percent of the population resides in urban localities (Gss & Macro, 2010).

The youth population (population less than 15 years) in the Municipality accounts for 31 percent of the population with a small number of elderly persons (population aged 65 years and older). The employed population represents 47.3 percent, of which 21.4 percent are engaged as skilled agricultural, forestry and fishery workers, 26.8 percent are engaged in service and sales, while 22.6 percent are into craft and related trade, and 15.8 percent are engaged as managers, professionals, and technicians (Gss & Macro, 2010).

### Study population

All male barbers in the Ho Municipality were eligible to participate in the study.

### Inclusion and exclusion criteria

Male barbers working in barbing shops who consented to be part of the study and those below 18 years who gave assent and whose parents/guardians consented, were included in the study. However, road-side barbers were excluded from the study. Road-side barbers were excluded because they are mobile barbers and are less likely to be members of barbers’ association. Therefore, they were not captured in the sampling frame used in sampling barbers for the current study.

### Study design

A cross-sectional descriptive design was used to determine the knowledge, attitudes and prevention practices of barbers regarding HIV in the Ho Municipality using structured questionnaires as the data collection tool. This design allowed for quick and easy data gathering even from a large target population.

The snapshot nature of cross-sectional studies, while convenient, is limited in that it does not provide a good basis for establishing causality. Three distinct variables were measured in the current study at the same point in time, but it could not positively be determined if one caused the other. This was however curbed in the study by decreasing bias in the data collection process as the questionnaire measured each variable separately and data analysis was done on the various variables to ascertain if there were associations between them.

### Sample size determination

The minimum sample size was obtained for this study by using the Cochran formula (Cochran, 1977),

$$n=\frac{Z^{2}pq}{d^{2}}$$

Where *n* = Sample size, Z = Z score, *p* = estimated prevalence of an attribute that is present in the population, *q* = 1-*p*, d = margin of error.

**Table:** 

*n* = sample size	*n*=?
*Z* = *Z*-score	*z* = 95% = 1.96
*P* = prevalence	*p* = 0.46
*q* = 1-prevalence	*q* = 1-0.46
*d* = Margin of error	*d* = 0.1

It was based on the assumption of a margin of error of 0.1, 95% confidence level and 0.05 (5%) non-response rate. The *P* = adequate knowledge regarding HIV/AIDS among barbers in a previous study conducted in Sokoto, Nigeria (46%) (Ibrahim, Opara, & Tanimomo, 2007); thus, the calculated sample size was *n* = 95.4.

$$A\;\mathrm{sample}\;\mathrm{size}\;(n)=\frac{{\left(1.96\right)}^{2}\times 0.46\times (1-0.46)}{{\left(0.1\right)}^{2}}$$

*n* = 95, Adding a non-response rate of 10%, *n* = (95*0.1) + 95 = 104.5.

Therefore, the minimum sample size for the current study was 105. However, the sample size used for the current study was 121.

### Sampling method

A multistage sampling was used to sample ultimate respondents. A municipality was divided into clusters within which three clusters were selected in the first instance and in the second stage, the barbers were asked to participate in the current study. A simple random sampling technique was used to sample the barbers from the barbers’ list of the Ghana National Association of Barbers, Ho Secretariat. A cluster represented a sub-municipality in the Ho Municipality. The names of the communities in each cluster were written on pieces of paper, folded and shaken to ensure they mixed well. Using the lottery method, three communities were selected randomly from each sub-municipality. Barbers were then selected randomly from each selected community using the sampling frame of barbers provided by the Ho Barbers’ Association, until the required sample size was reached. The 121 barbers in Ho were evenly distributed across all the clusters.

### Data collection

Data were collected using pretested structured questionnaires adapted from previous studies (Arulogun & Adesoro, 2009; Patel et al., 2015; Zewudie, Legesse, & Kurkura, 2002). To standardise the questionnaire further, Cronbach’s alpha was used to evaluate the reliability of the questions and items.

### Data analysis

Data were entered using Epi Data Software Version 3 and analyzed using Stata Version 14.0. Data were represented using tables and graphs; Chi-squared and Logistic regression analyses were conducted with 5% used to indicate level of significance to determine the association between the dependent variables (knowledge, attitude and prevention practices regarding HIV/AIDS) and independent variables (demographic characteristic like age, marital status, educational level). Knowledge was assessed using 7 items and was rated as good if a barber had 5 or more correct answers and poor if a barber had less than 5 correct answers. Attitude was assessed using 4 items and was dichotomised into good attitude and poor attitude. Anyone who responded ‘yes’ to 3 or more questions was considered as having good attitude. On the other hand, anyone who answered ‘yes’ to less than 3 questions, was considered as having poor attitude towards HIV/AIDS. Practices were assessed by rating them into good and bad practices. Anyone who answered more than 7 questions correctly was rated good, and poor if a barber answered less than 7 questions correctly.

A Cronbach’s alpha of 0.78 for knowledge regarding HIV/AIDS among barbers was obtained while that for prevention practice was 0.61 and that for attitude was 0.34. It is important to note that according to Polit and Beck (2004) the following characteristics of the measurement situation can affect the value of the Cronbach’s alpha obtained:

- The coefficient alpha does not provide a very good estimate when the items making up the measurement scale are heterogeneous in their relationship to each other or when their number is small. The more items the instrument contains the more accurate the alpha coefficient.
- The Cronbach alpha increases with the spread of variance of scores. Low reliability coefficient may also be due to the homogeneity of the sample. The more homogeneous the sample is, the lower is the Cronbach alpha coefficient.
- The alpha coefficient is a function of test length. The longer the test the higher the level of alpha. The Cronbach alpha is lower when a response with two possible answers was used. The coefficient is improved when a Likert scale response option is used.

The lower reliability coefficient observed for attitude could be due to these characteristics. Therefore, the Cronbach’s alpha showed that the combination of the questions for measuring level of knowledge, attitude and prevention practice had acceptable reliability based on Taber’s interpretation (Taber, 2018).

## Ethical issues

Ethical approval for the study was obtained from the Ghana Health Service Ethics Review Committee through the University of Health and Allied Sciences with approval number GHS-ERC: 31/05/17. Permission was sought from the Ho Municipal Health Directorate and the Ghana National Association of Barbers (GNAB) before the study was conducted. Participants were also assured that under no condition whatsoever would their names or any other contacts be allied to the data analysis and dissemination of the findings of the study. It was made clear to the participants that all their responses would be confidential during and after data collection. Furthermore, participants were assured that storage, analysis and reporting of all data including dissemination would be done in codes, hence identity of the respondents will not be exposed. In addition, a participant’s Informed consent and Assent sheets, which provided details and willingness to participate in the study were administered to the participants and they were required to designate their acceptance and approval to participate in the study.

## Results

### Demographic and occupational characteristics

Table 1 shows the demographic and occupational characteristics of the participants. The median age and the interquartile range of the participants was 25 (22–30), while the mean (S.D) was 26.5 (5.63) and the range was 18–47. Majority of the participants 81 (66.9%) were aged 20–29 years, 66 (54.6%) were single, 54 (44.6%) had secondary education and 96 (79.3%) were Christians. Majority, 59 (48.8%) had less than 5 years’ work experience, 113 (93.4%) had less than 5 assistants to work with, 53 (43.8%) attended to 10–19 clients per day, 93 (76.9%) were located in the urban area and 70 (57.9%) learnt the profession through apprenticeship.

**Table 1.** Demographic and occupational characteristics of the participants.

Variables	Frequency (*N* = 121)	Percent (%)
Median age (interquartile range)	25(22–30)	** **
*Age range*	18–47	** **
*Mean age (S.D)*	26.5 (5.63)	** **
*Age group (years)*		
<20	8	6**.**6
20–29	81	66**.**9
30+	32	26**.**5
*Marital status*		
Single	69	57**.**0
Married	39	32**.**3
Cohabiting	13	10**.**7
*Educational level*		
None	21	17**.**4
Primary	42	34**.**7
Secondary	58	47**.**9
*Religion*		
Christian	96	79**.**3
Muslim	20	16**.**6
Traditional	5	4**.**1
*Work experience*		
<5 years	59	48**.**8
5–10 years	43	35**.**5
>10 years	19	15**.**7
*Number of assistants per barber*		
<5 Assistants	113	93**.**4
>5 Assistants	8	6**.**6
*Number of clients attended to per day*		
<10	45	37**.**2
10–19	53	43**.**8
20+	23	19**.**0
*Location*		
Urban	93	76**.**9
Rural	28	23**.**1
*Mode of learning*		
Apprenticeship	70	57**.**9
Barbering school	6	5**.**0
On-the-job training	31	25**.**6
Others	14	11**.**5

### Knowledge on HIV/AIDS

Table 2 describes the knowledge on HIV/AIDS of the barbers. Majority, 114 (94.2%) had heard of HIV/AIDS and less than half, 58 (47.9%) knew HIV/AIDS was caused by a virus. Majority, 88 (72.7%) knew barbers were at risk of infecting clients and 80 (66.1%) also knew clients were at risk of infecting barbers. Majority, 76 (62.8%) knew HIV is preventable and 82 (67.8%) also knew sterilisation of barbing instruments could prevent HIV transmission. Of the 121 participants, most 77 (63.6%) had good knowledge on HIV/AIDS.

**Table 2.** Knowledge on HIV/AIDS of the participants.

Variables	Frequency (*N* = 121)	Percent (%)
*Heard of HIV/AIDS*		
Yes	114	94.2
No	7	5.8
*Causes of HIV*		
Germs	37	30.6
Virus	58	47.9
Witchcraft	14	11.7
Bacteria	6	4.9
Others	6	4.9
*Barber’s risk of infecting clients*		
Yes	88	72.7
No	14	11.6
Do not know	19	15.7
*Client’s risk of infecting barbers*		
Yes	80	66.1
No	22	18.2
Do not know	19	15.7
*HIV is preventable*		
Yes	76	62.8
No	22	15.8
Do not know	19	21.4
*Sterilisation can prevent HIV*		
Yes	82	67.8
No	7	5.7
Do not know	32	26.5
*Level of knowledge*		
Good knowledge	77	63.6
Poor knowledge	44	36.4

#### Association between knowledge regarding HIV/AIDS and demographic characteristics

Table 3 shows the association between participants’ knowledge regarding HIV/AIDS and demographic characteristics. Barbers with secondary education were more likely to have good knowledge regarding HIV/AIDS compared to those with no formal education [AOR = 10.22 (95% C.I: 2.24, 46.64) *p* = 0.003]. Barbers with 5–10 years’ work experience were more likely to have good knowledge regarding HIV/AIDS than those with <5 years’ work experience [AOR = 13.56 (95% C.I: 2.73, 67.25) *p* = 0.001]. Barbers with good attitudes towards HIV/AIDS were more likely to have good knowledge regarding HIV/AIDS than those with poor attitudes [AOR = 4.07 (95% C.I:1.27, 13.08) *p* = 0.018]. However, barbers who were cohabiting with their sexual partners were less likely to have good knowledge regarding HIV/AIDS than those who were single [AOR = 0. 07 (95% C.I: 0.01, 0.50) *p* = 0. 008]. In the same vein, barbers who had 10–19 clients a day were less likely to have good knowledge regarding HIV/AIDS than those who had <10 clients a day [AOR = 0.13 (95% C.I: 0.03, 0.52); *p* = 0.004].

**Table 3.** Association between demographic characteristics of Barbers and odds of knowledge.

Variables	Knowledge		Chi-square *X*^2^ (*p*-value)	^a^COR (95% CI) *p*-value	^b^AOR (95% CI) *p*-value
Age group	Good knowledge [*N* = 77]	Poor knowledge [*N* = 44]			
<20	5 (62.5)	3 (37.5)			
20–29	50 (61.7)	31 (38.3)		0.97(0.21, 4.33)0.966	1.18 (0. 15, 8. 80) 0.868
30+	22 (68.7)	10 (31.3)	4.27 (0.233)	1.3 (0. 26, 6.63) 0.736	1.09 (0. 09, 13.16) 0.944
*Marital status*					
Single	49 (71.0)	20 (29.0)			
Married	24 (61.5)	15 (38.5)		0. 65 (0. 28, 1.48) 0.310	0. 30 (0 .07, 1. 29) 0.108
Cohabiting	4 (30.8)	9 (69.2)	8.90 (0.031)	0. 20 (0. 06, 0.67) 0.010	**0. 07 (0.01, 0.50) 0. 008**
*Educational level*					
None	7 (33.3)	14 (66.7)			
Primary	27 (64.3)	15 (35.7)		3.43 (1.16, 10.09) 0.025	4. 61 (0. 95, 22.20) 0.057
Secondary	43 (74.1)	15 (25.9)	12.34 (0.006)	5.42 (1.88, 15.58) 0.002	**10.22 (2.24, 46.64) 0.003**
*Religion*					
Christian	63(65.6)	33(34.4)			
Muslim	13(65.0)	7(35.0)		0.97 (0.35, 2.67) 0.957	0.68 (0.20, 2.36) 0.552
Traditional	1(20.0)	4(80.0)	4.29(0.117)	0.13 (0. 01, 1.22) 0.074	0.20 (0.02, 1. 84) 0.158
*Work experience*					
<5 years	32 (54.2)	27 (45.8)			
5–10 years	33 (76.7)	10 (23.3)		2.78 (1.16, 6.66) 0.022	**13.56 (2.73, 67.25) 0.001**
> 10 years	12 (63.2)	7 (36.8)	5.44 (0.066)	1.44 (0.49, 4.18) 0.496	4.46 (0. 45, 43. 90) 0.200
*Number of apprentice per barber*					
<5 Assistants	73 (64.6)	40 (35.4)			
>5 Assistants	4 (50.0)	4 (50.0)	0.68 (0.407)	0. 54 (0. 13, 2.30) 0.412	0. 20 (0.02, 2.07) 0.179
*Number of client attendance per day*					
<10	35 (77.8)	10 (22.2)			
10–19	29 (54.7)	24 (45.3)		0.34 (0.14, 0.83) 0.019	**0.13 (0.03, 0.52)0.004**
20+	13 (56.5)	10 (43.5)	6.21 (0.045)	0.37 (0.12, 1.09) 0.073	0.30 (0.06, 1.47)0.139
*Location*					
Urban	60 (64.5)	33 (35.5)			
Rural	17 (60.7)	11 (39.3)	0.13 (0.714)	0.85 (0.35, 2.02) 0.714	0.33 (0.08, 1.23)0.101
*Mode of operation*					
Apprenticeship	45 (64.3)	25 (35.7)			
Barbering school	5 (83.3)	1 (16.7)		2.77 (0. 30, 25.11) 0.363	2.64 (0.03, 190.14) 0.655
On the job training	15 (48.4)	16 (51.6)		0.52 (0. 22, 1.22) 0.136	0.45 (0.13, 1.58) 0.218
Others	12 (85.7)	2 (14.3)	7.08 (0.069)	3.33 (0. 69, 16.09) 0.134	7.00 (0.58, 83.91) 0.125
*Attitude*					
Poor attitude	28 (56.0)	22 (44.0)			
Good attitude	16 (22.5)	55 (77.5)	14.19 (<0.001)	4.37 (1.98, 9.62) <0.001	**4.07 (1.27, 13.08) 0.018**

### Attitudes of barbers regarding HIV/AIDS

Table 4 shows the attitudes of barbers regarding HIV/AIDS. Majority, 87 (71.9%) had not tested for HIV/AIDS before. Of the 121 participants, majority, 83 (68.6%) agreed that they would attend to clients who are HIV-positive, 104 (85.9%) agreed that they would continue with their profession if they became HIV-positive, 50 (41.3%) were willing to attend to clients who disclosed their HIV status to them, while only 14 (11.6%) would attend to them but would have sterilised the instruments after use. Overall, majority, 71 (58.7%) portrayed a good attitude regarding HIV/AIDS.

**Table 4.** Attitude of Barbers regarding HIV/AIDS.

Variables	Number*****N* = [121]	Percentage (%)
*Having tested for HIV/AIDS before*		
Yes	34	28.1
No	87	71.9
*Attend to a client with HIV*		
Yes	83	68.6
No	38	31.4
*Continue your profession if you have AIDS*		
Yes	104	85.9
No	17	14.1
*Attitude towards a client who discloses his/her status to you*		
Welcome the person	50	41.3
Reject the person	23	19.0
Welcome, but will sterilise afterwards	14	11.6
Others	34	28.1
*Overall Attitude regarding HIV/AIDS*		
Good Attitude	71	58.7
Bad Attitude	50	41.3

#### Association between demographic characteristic and attitudes regarding HIV/AIDS

Table 5 shows the association between demographic characteristics and attitudes regarding HIV/AIDS. Likewise, barbers with good knowledge regarding HIV/AIDS were more likely to have good attitudes regarding HIV/AIDS than those with poor knowledge [AOR = 5.48 (95% C.I: 2.01, 14.93); *p* = 0.001]. However, barbers who had on-the-job training were less likely to have good attitudes regarding HIV/AIDS than those who were trained through apprenticeship [AOR = 0.32 (95% C.I: 0.11, 0.89); *p* = 0.029].

**Table 5.** Association between demographic characteristics of Barbers and odds of Attitude

Variables	Attitude		Chi-square X^2^ (*p*-value)	^a^COR (95%CI) *p*-value	^b^AOR (95%CI) *p*-value
Educational Level	Poor attitude [*N* = 50]	Good attitude [*N* = 71]			
No formal education	13 (61.9)	8 (38.1)			
Primary	13 (30.9)	29 (69.1)		3.47 (1.18, 10.14) 0.023	3.13 (0.88, 11.14) 0.077
Secondary	24 (41.4)	34 (58.6)	8.56 (0.036)	2.23 (0.82, 6.09) 0.115	2.02 (0.59, 6.82) 0.257
*Religion*					
Christian	38 (39.6)	58 (60.4)			
Muslim	10 (50.0)	10 (50.0)		0.65 (0. 24, 1.72) 0.392	0.59 (0.20, 1.74) 0.344
Traditional	2 (40.0)	3 (60.0)	0.74 (0.689)	0. 98 (0. 15, 6.15) 0.985	2.65 (0.31, 22.51) 0.371
*Work experience*					
<5 years	27 (45.8)	32 (54.2)			
5–10 years	20 (46.5)	23 (53.5)		0 .97 (0. 44, 2.13) 0.940	0.72 (0.28, 1.81) 0.489
> 10 years	3 (15.8)	16 (84.2)	5.44 (0.066)	4.5 (1.18, 17.10) 0.027	3.76 (0.78, 18.14) 0.098
*Mode of learning*					
Apprenticeship	23 (32.9)	47 (67.1)			
Barbering school	4 (66.7)	2 (33.3)		0.24 (0.04, 1.43) 0.119	0.20 (0.03, 1.18) 0.077
On-the-job training	20 (64.5)	11 (35.5)		0.26 (0.11, 0.65) 0.004	**0.32 (0.11, 0.89) 0.029**
Others	3 (21.4)	11 (78.6)	12.82 (0.005)	1.7 (0.45, 7.06) 0.403	0.40 (0.88, 1.81) 0.236
*Community of operation*					
Urban	42 (45.2)	51 (54.8)			
Rural	8 (28.6)	20 (71.4)	2.44 (0.118)	2.05 (0.82, 5.14) 0.122	1.90 (0.61, 5.84) 0.262
*Knowledge level*					
Poor knowledge	28 (63.6)	16 (36.4)			
Good knowledge	22 (28.6)	55 (71.4)	14.19 (<0.001)	4.37 (1.98, 9.62) <0.001	**5.48 (2.01, 14.93) 0.001**
*Practices*					
Poor practices	47 (43.9)	60 (56.1)			
Good practices	3 (21.4)	11 (78.6)	3.21 (0.073)	2.87 (0.75, 10.89) 0.121	0.59 (0.12, 2.72) 0.503

### Prevention practices of barbers regarding HIV/AIDS

Table 6 shows the prevention practices of barbers regarding HIV/AIDS. Among the 121 participants, the majority, 101 (83.5%) used razors and clippers on clients, 61 (50.4%) had frequent hand wash after a haircut and between clients, 76 (62.8%) used water and disinfectants to clean their instruments, 104 (85.9%) cleaned their instruments immediately after use on a client and 45 (37.2%) had frequent disinfection and hand washing after every client. Of the 121 participants, majority, 75 (62.0%) had UV lights and 61 (50.4%) could not remember how frequently they had changed their UV lights. Overall, majority, 106 (87.6%) exhibited bad HIV prevention practices.

**Table 6.** Prevention practices of Barbers regarding HIV/AIDS.

Variables	Number*****N* = [121]	Percentage****(%)
*Use of razors and clippers on clients*		
Yes	101	83.5
No	20	16.5
*Having frequent hand wash after every hair cut or between clients*		
Yes	61	50.4
No	60	49.6
*What you use in cleaning your instrument*		
Water and disinfectant	76	62.8
Cotton soaked in a disinfectant	24	19.8
Water and Soap	17	14.1
Others	4	3.3
*The time of cleaning tools for use*		
Immediately after use on one client	104	85.9
After use on several clients	17	14.1
Never clean them		
*Frequency of disinfecting/washing your hands*		
After every client	45	37.2
After more than 3 clients	18	14.8
After more than 5 clients	17	14.1
Not at all	40	33.1
Others	1	0.8
*Having a UV light in the barbing shop*		
Yes	75	62.0
No	46	38.0
*Frequency of changing the UV light*		
Weekly	4	3.3
Monthly	36	29.8
Yearly	20	16.5
Others	61	50.4
*Overall prevention practices regarding HIV/AIDS*		
Good Practices	15	12.4
Bad Practices	106	87.6

#### Association between prevention practices and demographic characteristics

Table 7 shows the association between practices and demographic characteristics. Barbers who had >10 years’ work experience were more likely to exhibit good HIV prevention practices than those with <5 years’ work experience [AOR = 24.92 (95% C.I: 2.08, 297.86); *p* = 0.011].

**Table 7.** Association between demographic characteristics of Barbers and odds of practices.

Variable	Practices		Chi-square *X*^2^ (*p*-value)	^a^COR (95%CI) *p*-value	^b^AOR (95%CI) *p*-value
Age group	Poor practices [*N* = 107]	Good practices [*N* = 14]			
<20	8 (100.0)	0 (0.0)			
20–29	74 (91.4)	7 (8.6)		1.71 (0.08, 32.67) 0.721	1.53 (0.06, 35.96) 0.790
30+	25 (78.1)	7 (21.9)	4.29 (0.231)	4.99 (0.25, 97.07) 0.288	0.72 (0.02, 24.84) 0.858
*Educational level*					
No formal education	19 (90.5)	2 (9.5)			
Primary	36 (85.7)	6 (14.3)		1.38 (0.29, 6.60) 0.679	0.41 (0.05, 3.02) 0.386
Secondary	52 (89.7)	6 (10.3)	0.87 (0.830)	0.96 (0.20, 4.54) 0.965	0.48 (0.08, 2.86) 0.423
*Work experience*					
<5 years	57 (96.6)	2 (3.4)			
5–10 years	39 (90.7)	4 (9.3)		2.92 (0.51, 16.74) 0.228	2.41 (0.42, 13.62) 0.317
>10 years	11 (57.9)	8 (42.1)	19.87 (<0.001)	20.72 (3.86, 111.05) <0.001	**24.92 (2.08, 297.86) 0.011**
*Mode of learning*					
Apprenticeship	61 (87.1)	9 (12.9)			
Barbering school	6 (100.0)	0 (0.0)		0.50 (0.03,9.58) 0.644	0.43 (0.01, 13.94) 0.635
On-the-job training	30 (96.8)	1 (3.2)		0.31 (0.53, 1.90) 0.207	0.48 (0.06, 3.83) 0.495
Others	10 (71.4)	4 (28.6)	5.24 (0.155)	2.77 (0.76,10.17) 0.124	0.57 (0.09, 3.54) 0.551
*Community of operation*					
Urban	86 (92.5)	7 (7.5)			
Rural	21 (75.0)	7 (25.0)	8.77 (0.003)	4.09 (1.29, 12.94) 0.016	3.97 (0.86, 18.20) 0.076
*Attitude*					
Poor	47 (94.0)	3 (6.0)			
Good	60 (84.5)	11 (15.5)	3.21 (0.073)	2.87 (0.75, 10.88) 0.121	0.42 (0.07, 2.42) 0.334
*Knowledge*					
Poor	43 (97.7)	1 (2.3)			
Good	64 (83.1)	13 (16.9)	3.92 (0.048)	8.73 (1.10, 69.24) 0.040	**12.57 (1.35, 116.86) 0.026**

Barber who had good knowledge on HIV/AIDS were more likely to exhibit good HIV prevention practices than those with poor knowledge [AOR = 12.57 (95% C.I: 1.35, 116.86); *p* = 0.026].

## Discussion

This cross-sectional study investigated the knowledge, attitudes and prevention practices among barbers in Ho, Ghana, regarding HIV/AIDS. The transmission of HIV/AIDS is usually considered through blood transfusions, sexual contacts, MTCT and use of HIV-infected instruments. However, barbing is considered among the least major routes of HIV transmission. Although barbers do not carry out procedures that deliberately penetrate the skin, the procedures can inadvertently damage the skin through abrasion or minor accidental cuts (Chanda & Khan, 2004). In relation to this finding, barbers have not been given the needed attention to curb the spread of the disease. Barbing has become a means of livelihood for the unemployed in the Ho municipality due to the high unemployment rate in the country; however, there is little or no training on the standard protocol for barbing.

This study showed that the majority of the barbers (63.6%) had an overall good knowledge on HIV/AIDS and 58.7% showed good attitudes regarding HIV/AIDS. However, majority (87.6%) manifested poor HIV prevention practices. The level of knowledge of HIV/AIDS of 63.6% is not in agreement with the Ghana demographic and health survey (GDHS), which claimed 100% knowledge of HIV/AIDS (GSS, GHS & International ICF, 2010). This difference could be in terms of the sample size used, literacy rate and population size in the GDHS. The GDHS data were obtained from the sentinel sites only. The GDHS targets the general population and therefore, a larger sample size than that of the current study, which focuses on barbers who may have a lower level of education than participants of the GDHS. The fact that the HIV prevalence in Ghana is consistently higher among at-risk groups such as commercial sex workers, clients at STI clinics and long-distance truck drivers and as such the awareness, education and prevention is geared towards them. These sub-populations with higher prevalence and risk of transmission constitute a reservoir for sustaining an epidemic (Addo et al., 2014).

A study conducted by Zewudie et al. (2002) in South-Western Ethiopia, reported that only 51.0% of barbers knew that HIV could be transmitted through barbing equipment, which is lower than the 63.6% found in the current study. This difference could be due to the differences in the sample size, time of the study, prevalence of HIV/AIDS and geographical location between the two studies. However, the 63.6% good knowledge regarding HIV/AIDS as reported in the current study could be considered as inadequate. Knowledge regarding HIV should be universal and 100% in order to effectively tackle the disease.

The significant association between educational level and level of knowledge found in the current study is in accordance with a study conducted by Akumiah and Sarfo (2015) in Obuasi, Ghana, which revealed that the knowledge of the barbers increases with increasing educational level. This is also similar to a descriptive cross-sectional study conducted by Adoba et al. (2015), in Obuasi in the Ashanti Region, Ghana, where it was discovered that 52.2% of the barbers who had knowledge on viral infections had a post-secondary education. This could decrease the transmission of viral-borne diseases as a result of barbers having knowledge of good sterilisation and disinfection practices.

The current study also found a significant association between barbers’ knowledge and work experience. This is contrary to the observations by Wazir, Mehmood, Ahmed, and Jadoon (2008) in Pakistan, in which the level of knowledge among barbers about health hazards associated with their years of profession was found to be very poor. The reason for this discrepancy could be the demographic differences between the study area in Pakistan and Ho. Barbers in Ho are usually educated on HIV/AIDS by their barbers’ association and therefore, barbers with a longer work experience are more likely to have a better knowledge on HIV/AIDS than those with a shorter work experience. This might not be the case in Pakistan.

Most of the participants in this current study did not know their HIV/AIDS status and would continue their profession if they were infected. This could pose a major risk to clients who patronise these barbers. Hence, more awareness should be advocated to get the barbers to know their status and observe proper adherence to prevention practices in order to minimise the chances of HIV transmission.

The attitudes of the participants were generally favourable as the majority of barbers had good attitudes regarding HIV/AIDS (58.7%). This is in accordance with a previous study conducted in South-Western Ethiopia, which revealed that the majority of barbers had favourable attitudes regarding HIV/AIDS (67.8%) (Zewudie et al., 2002). The favourable attitudes of the barbers in the current study may be due to their access to adequate information in their mode of learning the barbing profession and knowledge on disease transmission caused by unsterile sharp equipment. However, it is worth noting that the overall 58.7% good attitude reported in this study is inadequate with respect to HIV/AIDS. To effectively tackle HIV/AIDS, there should be 100% good attitude towards the disease. Poor attitudes towards HIV/AIDS could lead to stigma and discrimination against PLHIV. Similarly, with knowledge, the current study showed significant associations between attitude and work experience and between attitude and level of knowledge. These positive associations could result from the significant association between knowledge and attitude as found in the current study, with barbers having good knowledge regarding HIV/AIDS more likely to have good attitudes towards HIV/AIDS.

Barbers in the current study manifested poor prevention practices regarding HIV/AIDS. This study demonstrated that barbers in the study area seemed to be practicing disinfection instead of sterilisation. Micro-trauma induced while shaving causes release of blood and other bodily fluids, which can cause transmission of HIV to barbers when they come in contact with these fluids. Also, contamination of the shaving instruments can pose a great risk to other clients. Despite this, few barbers in the current study were aware of the risk posed by unsafe shaving practices and the mode of transmission of HIV/AIDS infections. Most of the barbers were found to have UV lights in their barbershops but the bulbs were not frequently changed. This is in agreement with a study conducted in Obuasi, Ghana which indicated that a UV radiation steriliser cabinet was seen in all barbershops visited, but the majority of the sterilisers could only be described as storage cabinets or used for display purposes. Furthermore, most of the steriliser cabinets either used mercury bulbs instead of UV bulbs or did not have any light source in them (Akumiah & Sarfo, 2015).

The reason for these flawed professional practices could be attributed to lack of information about the sterilisation process. Most barbers were seen placing their hair trimmers into the steriliser cabinets when not in use or after use on a client. But for those barbershops with heavy workload the same set of hair trimmers could be used continuously for multiple clients without sterilisation (Mutocheluh & Kwarteng, 2015).

Skin damage is the prerequisite for inoculation of the scalp with HIV. It allows for the penetration of the skin with barbing instruments and exposes the circulatory system to infection. Skin damage occurs during barbing either as an accidental cut or abrasion as a result of blade-to-skin contact. Blade-to-scalp contact often occurs during hair shaping, shaving and zero-hair cutting involving use of a detachable plastic comb (Arulogun & Adesoro, 2009).

In the current study, majority of participants were aware of HIV/AIDS, but lacked adequate sterilisation practices and could not associate their practices to the risk of transmission. This is in accordance with studies carried out in Morocco, Ethiopia and Pakistan, which showed that the level of knowledge, awareness and practices of barbers about the concept of infectious-risk associated with blood was generally very low (Khuwaja, Qureshi, & Fatmi, 2002). It is also in accordance with the study by Belbacha, Cherkkaoui, Akrim, Dooley, and El Aouad (2011), which found that traditional barbers and their clients in Morocco are unfamiliar with proper practices and are mostly unaware of the transmission of blood borne pathogens through shaving tools.

For these reasons, awareness campaigns are imperative and should focus on both barbers and the general population especially those who are at risk due to their occupation. Also, for other viral infections easily transmitted through the barbing process, such Hepatitis B and Hepatitis C, training and vaccination should be encouraged in order to curb the increasing incidence of these viral blood borne diseases (Arulogun & Adesoro, 2009).

A possible reason for the poor HIV-prevention practices as reported in the current study may be the lack of strict control measures and monitoring by relevant bodies. Unlike in developed countries where activities of barbers are regulated through a comprehensive training, licensing and monitoring programs, barbers in Ghana have not been given any noticeable attention to their profession and their activities (Mutocheluh & Kwarteng, 2015).

Majority of the barbers with poor practices were found to be to aged 20–29 years. This is in agreement with a cross-sectional study conducted by Dongdem et al. (2012), to estimate the prevalence of HIV/HBV/HCV among blood donors at the Tamale Teaching Hospital in Ghana, which observed that the highest prevalence of HIV/HBV/HCV were among donors within the ages of 20–29. This supports findings from this study that barbers’ practices could serve as a major route of transmission of HIV/AIDS. Barbers with >10 years’ work experience were more likely to exhibit good prevention practices as compared to those with <5 years’ work experience.

## Limitations

Most participants were reluctant to participate in the study because they thought that they were going to be sanctioned if it was found that they were not licensed to practice. Those who also consented to participate were afraid that if their poor practices were revealed, the public health authorities would halt their operations. Participants could have answered the questions to their advantage since observation was not used as a tool to collect data because the study design was quantitative. Furthermore, we acknowledge that with our limited sample the true effect of significant findings is uncertain and endeavor to further investigate this HIV prevention practices in this setting.

## Conclusion

In conclusion, barbers involved in the study had relatively good knowledge and attitudes regarding HIV/AIDS, though these could be considered inadequate, and also exhibited poor prevention practices regarding HIV/AIDS. Knowledge was influenced by level of education, work experience and number of clients a day, while attitude was influenced by mode of training and level of knowledge. Practice was influenced by work experience. Programmes aimed at improving the knowledge, attitudes and practices should be implemented among barbers, with a specific focus on those without any formal education, those with less than five years work experience, those who had on-the-job training and those with more than ten clients a day. These programming initiatives may contribute to curbing new HIV infections.

## Footnotes

^a^
Crude Odds Ratio.

^b^
Adjusted Odds Ratio.

^a^
Crude Odds Ratio.

^b^
Adjusted Odds Ratio.

^a^
Crude Odds Ratio.

^b^
Adjusted Odds Ratio.
